# Automated Insulin Delivery Systems in Pediatric Type 1 Diabetes: A Narrative Review

**DOI:** 10.1177/19322968241248404

**Published:** 2024-05-24

**Authors:** Peter Adolfsson, Ragnar Hanas, Dessi P. Zaharieva, Klemen Dovc, Johan Jendle

**Affiliations:** 1 Institute of Clinical Sciences, Sahlgrenska Academy, University of Gothenburg, Gothenburg, Sweden; 2 Department of Pediatrics, The Hospital of Halland Kungsbacka, Kungsbacka, Sweden; 3 Department of Pediatrics, NU Hospital Group, Uddevalla, Sweden; 4 Division of Endocrinology, Department of Pediatrics, School of Medicine, Stanford University, Stanford, CA, USA; 5 Faculty of Medicine, University of Ljubljana, Ljubljana, Slovenia; 6 Department of Pediatric Endocrinology, Diabetes and Metabolic Diseases, University Children’s Hospital, Ljubljana, Slovenia; 7 School of Medicine, Institute of Medical Sciences, Örebro University, Örebro, Sweden; 8 Diabetes Endocrinology and Metabolism Research Centre, Örebro University, Örebro, Sweden

**Keywords:** automated insulin delivery, exercise, hybrid closed loop, pediatric, illness, type 1 diabetes

## Abstract

This narrative review assesses the use of automated insulin delivery (AID) systems in managing persons with type 1 diabetes (PWD) in the pediatric population. It outlines current research, the differences between various AID systems currently on the market and the challenges faced, and discusses potential opportunities for further advancements within this field. Furthermore, the narrative review includes various expert opinions on how different AID systems can be used in the event of challenges with rapidly changing insulin requirements. These include examples, such as during illness with increased or decreased insulin requirements and during physical activity of different intensities or durations. Case descriptions give examples of scenarios with added user-initiated actions depending on the type of AID system used. The authors also discuss how another AID system could have been used in these situations.

## Introduction

Achieving optimal glucose management while minimizing hypoglycemia remains significant challenges among youth with type 1 diabetes. Recommendations regarding clinical targets for continuous glucose monitoring (CGM) interpretation have been proposed, including time in range (TIR) and time below range (TBR).^1^

Diabetes technology has evolved markedly over the last two decades, and rapid progress is ongoing. Education, training, and appropriate use of diabetes technologies are cornerstones in achieving the proposed targets for optimal glycemic control.^2^ In adults with type 1 diabetes, CGM is considered a standard of care and an essential part of diabetes management as it improves long-term glucose control up to 36 months, regardless of the treatment modality.^3^ The development of diabetes technologies and clinical outcomes linked to these devices have led to changes in the recommendations for youth with diabetes, recently published in the International Society for Pediatric and Adolescent Diabetes (ISPAD) guidelines.^4^ When target glucose settings are discussed, new goals are also mentioned in terms of fasting glucose values between 3.9 and 7.8 mmol/L (70-140 mg/dL),^5^ and among younger children under the age of 7, the use of time in tighter range (TITR) between 3.9 and 7.8 mmol/L (70-140 mg/dL) have been recommended where at least 50% of the CGM values should be within this tighter target or at least 70% within a glucose TIR of 3.9 to 10 mmol/L (70-180 mg/dL).^6^ When considering this shift to TITR or additional glycemic targets, it is essential to approach this change with a patient-centered focus and consider the potential psychosocial impact of a narrower target glucose range in persons with type 1 diabetes (PWD). With disparities in the current guidelines, additional research in this area is warranted.

At the same time, some efforts are needed to address the potential threats from longstanding diabetes including possible brain damage and Alzheimer’s disease-related neurodegeneration.^7^ In a recent publication, an automated insulin delivery (AID) system was shown to increase specifically TITR, when settings were adjusted to the target of 5.5 mmol/L (100 mg/dL) and 2 hours of active insulin with the MiniMed 780G system (Medtronic, Northridge, CA, USA).^8^

In Sweden, TITR has been used in pediatrics for more than 10 years, along with a lower HbA1c target of 48 mmol/mol (6.5%) since 2017, contributing to a lower national mean HbA1c of 51.6 mmol/mol (6.85%) in 2022.^9^ A lower HbA1c target may generally motivate caregivers and families to make the most of their technology. In general, both CGM and AID systems are recommended to reduce the risk of hypoglycemia,^10^ and to improve glucose control.^11^ When CGM was added to continuous subcutaneous insulin infusion (CSII) or multiple daily injections (MDI), user-initiated actions by the PWD were needed to optimize glucose control. The first AID system was created when the CGM formed one part of a system where the other part, an insulin pump, was equipped with an algorithm that automatically adjusted the pump’s basal rate. With an AID system, a new decision is made about the insulin dose, up to 288 times per day, regardless of whether it is day or night. Doses are increased if the glucose level rises and vice versa if it falls. Furthermore, basal insulin doses can be automatically stopped in the face of impending hypoglycemia. Results later showed that AID systems significantly improved glucose control more than what could previously be achieved—both day and night, and with a lower diabetes burden.

Among children and young PWD, the efforts to reach optimal glycemic outcomes are critical, given the metabolic memory and the risk of deteriorated glucose control, long-term diabetes complications and the increased mortality that may otherwise occur in the future.^12^

This narrative review examines the current AID utilization in pediatric type 1 diabetes, focuses on recent research findings, challenges, and potential opportunities. The review is limited to the following AID systems: CamAPS FX^TM^ (CamDiab Ltd. Cambridge, UK), MiniMed 780G^TM^ (Medtronic, Northridge, CA, USA), Omnipod 5^TM^ (Insulet, Acton, MA, USA), and Tandem t:slim X2 with Control-IQ technology^TM^ (CIQ / Tandem, San Diego, CA, USA), Table 1.

**Table 1.** Overview of four AID Systems and Some of the Specific Functions.

System name	CamAPS FX	MiniMed 780G	Omnipod 5	Tandem t:slim X2 CIQ
Algorithm	CamAPS FX	SmartGuard	SmartAdjust	Control IQ
Compatible CGM	Dexcom G6 Libre 3	Guardian Link 3	Dexcom G6	Dexcom G6Dexcom G7
		Guardian Link 4		Libre 2 Plus
		Simplera Sync		
Algorithm Target Value	5.8 mmol/L Adjustable at different times of day and night, 4.4-11.0 mmol/L	5.5, 6.1, or 6.7 mmol/L	Predictive target value (60 minutes): 6.1, 6.7, 7.2, 7.8, or 8.3 mmol/L	Predictive target interval (30 minutes): 6.2-8.9 mmol/L Target value: 6.1 mmol/L
Higher Target Value	Adjustable	8.3 mmol/L	8.3 mmol/L	7.8-8.9 mmol/L
System Specifications	Ease off / Makes the algorithm more ‘relaxed’: Reduces insulin delivery considerable Stops insulin delivery if glucose < 7.7 mmol/L Raises glucose target temporarily (+2.5 mmol/L) Boost / Makes the algorithm more ‘responsive’: Increases insulin delivery by ~35% Once glucose reaches target, boost will not push glucose lower than target.	Enabling 0.025U bolus increments will facilitate more fine-tuning with young children and small insulin doses. Enabling faster bolus delivery will facilitate reduced postprandial hyperglycemia if bolus dose is activated a bit late.	If a correction bolus for hyperglycemia is added, the PWD should be recommended to use the SmartBolus calculator by tapping “Use Sensor” within the bolus calculator. The CGM trend is then included in its bolus calculation. By this, the SmartAdjust technology can add up to 30% more insulin to a suggested bolus to address hyperglycemia.	The optional Sleep Activity mode allows a more aggressive range of 6.1-6.7 mmol/L. The optional Exercise Activity means a decreased dose if glucose values are predicted to be less than 7.8 mmol/L and stops if predicted lower than 4.4 mmol/L Six different profiles are possible to preset and use at situations with shifting insulin requirements including individualized basal doses, ICR and ISF.
FDA Approval		Age ≥7 years	Age ≥2 years	Age ≥6 years
CE-Mark (EU)	Age ≥1 years	Age ≥7 years	Age ≥2 years	Age ≥6 years
Therapeutic Goods Administration (TGA)	Age ≥1 years	Age ≥7 years		Age ≥6 years

Although the AID systems have similarities in frequently adjusting insulin delivery based on continuously generated glucose values, the AID systems work differently in many aspects.

Therefore, training from health care professionals (HCPs) is required for the PWD for optimal use of these AID systems, including specific differences that exist between these various systems. Studies have revealed that unannounced meals^13,14^ and situations involving rapid changes in insulin requirements are a challenge.^15^ The AID systems offer some user-initiated adjustments, which can be crucial for overcoming rapidly changing insulin resistance. For example, these challenges may exist during an acute illness, such as a viral infection with a fever associated with increased insulin resistance. While on the other hand, gastroenteritis accompanied by reduced energy intake is often associated with decreased insulin resistance. In addition, exercises of different intensities and durations can also pose challenges for an AID system.

In this review, different cases will exemplify how the various AID systems can be used to achieve optimal glycemic outcomes in everyday life and during challenges such as acute illnesses or around physical activity.

## Method

A literature search was conducted on January 21, 2024, in PubMed for articles published between January 2018 and December 2023, using keywords “Artificial pancreas” OR “Closed-loop” OR “Automated Insulin Delivery” AND “Type 1 diabetes mellitus” AND “Children” AND “Randomized OR Randomized controlled trial,” for reports in English only. We limited our analysis to studies of single hormone AID systems 24 h per day that were of 3-month duration or longer. Studies describing mean glucose values, brain development, and C-peptide were excluded. The remaining studies found are shown in Table 2.

**Table 2.** References From the Literature Search.

Author + Reference no	Population children/adults/mix age (range) number (n)	AID system	Trial design	Study duration
Jill Weissberg-Benchell et al^16^	Mix age 6-83 years n=275 adults n=165 children	iLet Bionic Pancreas	RCT	13 weeks
Régis Coutant et al^17^	Children 6-12 years n=60	MiniMed 780G	RCT	72 weeks
R Paul Wadwa et al^18^	Children 2-6 years n=102	Tandem CIQ	RCT	13 weeks
Julia Ware et al^19^	Children 2-6 years n=25	CamAPS FX	RCT cross-over Fiasp vs Iasp	16 weeks
Mercedes J Burnside et al^20^	Mix 7-70 years n=94	CamAPS FX	RCT	24 weeks
Satish K Garg et al^21^	Mixed 2-80 years n=151	MiniMed 670G	RCT	6 months
Laurel H Messer et al^22^	Children 6-17 years n=165	iLet Bionic pancreas	RCT	26 weeks
Anthony Pease et al^23^	Mixed 12-25 years n=NA (simulations)	MiniMed 670G	HE analysis	3-6 months
Alison Roberts et al^24^	Mixed 12-25 years n=17	MiniMed 670G	RCT	6 months
Barbara Kimbell et al^25^	Children 1-7 years n=74	CamAPS FX	RCT	16 weeks
Thekla von dem Berge et al^26^	Children 2-14 years n=38	MiniMed 780G	RCT	20 weeks
Julia Ware et al^27^	Children 6-18 years n=133	CamAPS FX	RCT	6 months
Dulanjalee Kariyawasam et al^28^	Children 6-12 years n=21	Diabeloop for Kids	RCT cross-over	13 weeks
Erin C Cobry et al^29^	Children 6-13 years n=49	Tandem CIQ	RCT	16 weeks
Julia Ware et al^30^	Children 1-7 years n=74	CamAPS FX	RCT	32 weeks
Eric Renard et al^31^	Children mean age 8.6 years n=122	Tandem CIQ	RCT	18 weeks
Michael A Tsoukas et al^32^	Children Mean age 15.3 years n=135	MiniMed 630G Fiasp + pramlintide	RCT	6 months
Korey K Hood et al^33^	Mixed 14-29 years n=113	MiniMed 780G	RCT cross-over	28 weeks
Melissa J Schoelwer et al^34^	Children 6-13 years n=100	Tandem CIQ	RCT	16 weeks
Olivia J Collyns et al^35^	Mixed 7-80 years n=60	MiniMed 780G	RCT	12 weeks
Richard M Bergenstal et al^36^	Mixed 14-29 years n=113	MiniMed 780G	RCT cross-over	24 weeks
Erin C Cobry et al^37^	Children 6-13 years n=101	Tandem CIQ	RCT	44 weeks
Lauren G Kanapka et al^38^	Children 6-13 years n=101	Tandem CIQ	RCT	12 weeks
Elvira Isganaitis et al^39^	Mixed 14-24 years n=63	Tandem CIQ	RCT	6 months
Marc D Breton et al^40^	Children 6-13 years n=101	Tandem CIQ	RCT	16 weeks

## AID Summarized Results, Challenges and Opportunities in Youth With Diabetes

### Summarized Results

A total of 24 randomized trials met these criteria. In summary, AID systems were associated with improved glycemic outcomes including reduced HbA1c,^21,22,26,27^ improved TIR,^17,18,20,30-32,34,35,38-40^ reduced time below range (TBR),^21,28,36^ and time above range (TAR),^36^ as reflected in the reviews published in 2023.^41,42^ These effects were seen regardless of the type of insulin,^19^ in different age ranges, below 6-7 years or 7-17 years,^18,22,26,30,34,38^ and regardless of baseline glycemic control.^43^

More significant benefits were seen among PWD, with elevated HbA1c levels above 86 mmol/mol (10%) at baseline.^43^

However, the benefits of AID technology are more extensive than the improved overall glycemic control. Generally, the nocturnal period is most positively impacted by AID systems, with improved nocturnal TIR and decreased TBR.^17,18,20,30-32,34,35,38-40^ Consequently, sleep is also improved for many parents of children with diabetes,^37,29^ which is of significant clinical importance.

Furthermore, parameters reflecting psychosocial well-being are often enhanced based on patient reported outcomes^33,37^ including increased acceptance,^16^ independence,^24,25^ reduced emotional burden,^16,33^ and anxiety.^24^

### Challenges

There are several challenges related to diabetes management in youth. Following the onset of diabetes, insulin needs are often low among preschool children. During school age, insulin requirements vary depending on several factors, such as level of physical activity, illness, and stress. The daily variation in glycemia is evident.^44^ During puberty, the insulin requirements change noticeably and sometimes with hormonal influence as in the dawn phenomenon^45^ and menstruation.^46^ The main physiological challenge an AID system faces is keeping up with the rapidly changing insulin requirements.

### Opportunities

For most PWD, AID systems offer an opportunity for improved glucose control, but it is important to emphasize that knowledge of the system is essential for both HCPs and the PWD. Some manual inputs are required with each system, such as adjusting target glucose values, Insulin Sensitivity Factor (ISF) and Insulin Carbohydrate Ratios (ICR), the time of insulin action, bolus speed, bolus steps, and using different predefined profiles, where possible.

Furthermore, the amount of carbohydrates (CHO) consumed should still be announced to the system before the meal is consumed to optimize glycemia. However, the iLet pump is a recently approved AID system in the United States that does not require the PWD to enter the amount (grams) of CHO consumed, but rather to announce the meal (eg, breakfast, lunch, or dinner and usual for me, more, or less for that meal type).

In the future, we also believe that adequate sleep will be further emphasized as a treatment goal since sleep affects the risk of developing cardiovascular complications and impacts overall quality of life.^29,37^ Sleep evaluation regarding quantity and quality should regularly be performed among PWDs and their parents/caregivers.

Overall, AID use can help to increase the time set aside for various other tasks throughout the day.

## AID Summarized Differences

Generally, each AID system works similarly to, but there are some key differences worth noting. It is essential to understand these differences as they may offer opportunities to modify and further optimize overall glycemic outcomes.

AID Challenges and Opportunities With Increased Insulin Requirements During Illnesses

The challenge of any AID system is to adapt to a situation with a rapid change in glycemia and insulin sensitivity. For example, elevated glucose levels and ketones (preferably checked by blood ketones), may be the result of increases in insulin resistance caused by an acute infection or possibly by a pump site failure (eg, occlusion). In either case, a manual dose of insulin should be given with an insulin pen or syringe, and the pump site should be changed. For additional details, a table with insulin dose recommendations depending on ketone levels was published in the ISPAD Clinical Practice Consensus Guidelines 2018: Sick day management in children and adolescents with diabetes for suggested actions.^47^

Different approaches to increasing the amount of insulin depending on the type of AID system used are described below.

### CamAPS FX

TheCamAPS FX system works by setting extended boluses while setting the basal rate to zero. No immediate extra boluses are given. The glucose target is customizable in 30-minute segments across 24 h from 4.4 to 11.0 mmol/L (80-198 mg/dL). The predictive algorithm calculates the insulin requirements for the next 2.5 to 4 h, and insulin delivery is adjusted every 8 to 12 minutes.

The algorithm will respond with increased insulin dosage caused by the increased insulin resistance after 2.5 to 4 hours. A decrease in ICR will give more insulin for meals, but ISF cannot be changed in automated mode. Manual corrections can be given via the bolus calculator, entering zero CHO. The system learns from previous insulin delivery and meal bolus information to adapt to daily, day-to-day, and post-meal insulin needs.

If the user is unsatisfied with the amount of insulin, an alternative lower glucose target value can be entered to increase the dose besides using a “Boost” feature, meaning that the algorithm will deliver approximately 35% more insulin if hyperglycemia is experienced. Interestingly, the “Boost” function will stop when the algorithm predicts that the glucose level will reach the chosen glucose target value. Thus, two additional options exist to increase insulin doses.

### MiniMed 780G

For the algorithm with the MiniMed 780G, the total daily insulin dose (TDD) and ISF are based on the actual insulin delivered every 24 hours. The insulin pump can only work with one calculated ISF during each 24 h period. The same target glucose level is used during the day and night.

Auto-corrections are given every 5 minutes when the max basal is reached and sensor glucose (SG) is > 6.7 mmol/L (120 mg/dL). These will be delivered a maximum of 12 times/h and 8% of TDD during 45 minutes. The system targets 6.7 mmol/L (120 mg/dL) for corrections and uses a model-based prediction to estimate if a correction bolus will result in a glucose of < 4.4 mmol/L (79 mg/dL) in the next 2 hours. A decrease in ICR will give more insulin for meals, but ISF cannot be changed in automated mode. Manual corrections may be provided by entering zero carbs with a bolus. The pump then calculates if it thinks that an extra bolus is appropriate.

If the user is unsatisfied with the amount of insulin recommended, the PWD may enter an adjusted amount of CHO into the pump to get a suggestion of the dose that is desirable at the time, although this strategy is not recommended by the manufacturer.

### Omnipod 5

Omnipod 5 AID system is tubeless. The SmartAdjust™ technology considers the person’s TDD, and for optimal start, an accurate setting of the same with an appropriate dose to create stable glucose control is important. Omnipod 5 modulates insulin every 5 minutes using the user’s customized target glucose between 6.1 and 8.3 mmol/L (110-150 mg/dL). Based on a prediction of the glucose values 60 minutes ahead, the system increases, decreases, or pauses basal insulin delivery every 5 minutes. An adaptive basal rate is calculated automatically with every Pod change (every 72 hours) to adjust TDD to shifting insulin requirements. Besides the adaptation to the insulin requirement of the last Pod, the system uses a decaying weighted average of TDD and more heavily weights the previous four to five Pods when calculating the new adaptive basal rate. The system can deliver up to four times the adaptive basal rate to address a possible episode of hyperglycemia.

Target glucose values are adjustable from 6.1 to 8.3 mmol/L (110-150 mg/dL) in 0.5 mmol/L (10 mg/dL) increments for different times of the day, and if needed, a lower target value could be entered to receive larger insulin doses. Furthermore, if a correction bolus for hyperglycemia is, it is recommended that the PWD use the SmartBolus calculator by tapping “Use Sensor” within the bolus calculator.

The CGM trend is then included in the bolus calculation. By using this feature, the SmartAdjust™ technology can add up to 30% more insulin to a suggested bolus to address hyperglycemia.

### Tandem Control-IQ

With Tandem Control-IQ, the basal rate will be increased with higher predicted glucose values. However, the quickest way to increase insulin dosages is to switch to another personalized profile. In total, there are six possible alternative profiles) where the individual can use different preset profiles in the form of different basal rates, ICR, and ISF.

Thus, meal bolus and automated correction doses are increased besides an increased preset basal dose. Many users practice using a plus 30%, 50% and 100% profile with settings that also allow increasingly higher meal doses and correction doses. Regardless of the profile, Control-IQ technology will continue with the same predictive algorithm where the estimated glucose value 30 minutes ahead in time controls the dosage every 5 minutes. If needed, a correction dose equivalent to 60% of the dose calculated via the set ISF is delivered up to once per hour if the value in 30 minutes is predicted to exceed 10 mmol/L (180 mg/dL). The manufacturer recommends different profiles with different aggressive settings instead of using “fake” CHO.

## AID Challenges and Opportunities With Decreased Insulin Requirements During Acute Illnesses

With gastroenteritis, for example, the insulin requirements may decrease due to a limited CHO intake that may alsoraise ketone bodies (ie, “starvation ketones”). The insulin doses usually need to be decreased to avoid hypoglycemia. However, if the doses are decreased below the actual insulin requirements, ketones (preferably check blood ketones) may develop as a sign of insulin deficiency (ie, “deficiency ketones”). Any CHO intake is urgently needed (eg, sugary drink or candy), and small insulin doses can be given manually with the pump when SG has risen enough, approximately above 7 mmol/L (126 mg/dL) With an AID system, the user can adjust the bolus dose accordingly, but if the basal rate is not decreased enough, it may be better to switch to manual mode and set a temporary basal rate at approximately −50% to avoid insulin deficiency. With automated mode and hypoglycemia, it is important not to overtreat with CHO as this may cause the pump to increase the insulin delivery too much, causing recurrent episodes of hypoglycemia.

### CamAPS FX

The predictive algorithm will probably decrease insulin delivery quickly since it calculates the requirements for the next 2.5 to 4 h and adjusts insulin every 8 to 12 minutes. An alternative glucose target can be used, in addition to different target values during various time intervals across all hours of the day. Besides this, the function “Ease off” can be added to reduce the risk of hypoglycemia. The “Ease off function has numerous effects on the control algorithm, including (1) increased glucose target by 2.5 mmol/L, (2) increased insulin sensitivity by 50%, and (3) suspended insulin delivery below a higher glucose threshold (7.7 mmol/L; 140 mg/dL).

It can be set for up to 24 h or planned to start at a specific time (ie, planned Ease-off).

### MiniMed 780G

The basal rate will be lowered or stopped with lower glucose values, but the sensitivity will be changed only once per 24 hours. A temporary target of 8.3 mmol/L (150 mg/dL) can be used for more cautious insulin delivery as this prevents automated boluses. A temporary target can be set for a duration of time. With low glucose values, the pump may not suggest any meal bolus. Confirming these 0 U doses is still crucial as it will prepare the AID system for more active insulin dosing once the glucose rises. With starvation ketones, the best solution may be to enter CHO after approximately 10 minutes when the SG has started to rise and get an adequate insulin dose, although more research is warranted. Enabling 0.025 U bolus increments will facilitate more fine-tuning with young children and small insulin doses. The glucose target can be increased to 6.1 or 6.7 mmol/L (110 or 120 mg/dL) for less aggressive insulin dosing.

If frequent episodes of hypoglycemia occur, it may be better to turn off automated mode and switch to manual mode, where predicted low glucose suspension (PLGS) will be active as long as the sensor is functioning. A reduced temporary basal rate can then be activated if needed.

### Omnipod 5

A higher glucose target value can be used for gentler insulin administration. Additionally, the Omnipod 5 system’s “Activity feature” can be activated to reduce the amount of insulin delivered during exercise or, in this case, when reduced insulin delivery may be desired.

When the “Activity feature” is activated, the glucose target of 8.3 mmol/L (150 mg/dL) is used along with an adaptive basal rate.

Furthermore, the bolus calculator includes both CGM values and trend information to calculate the bolus doses.

### Tandem CIQ

The basal rate will be lowered or stopped with predicted lower glucose values. However, the quickest ways to decrease insulin dosages is to switch to another profile with a lower basal rate and gentler insulin settings with: higher ICR and correction factors (ISF). The algorithm will then use these settings for the basal rate, meal boluses, or correction boluses. If the additional “Exercise mode” is activated, it will decrease the basal rate if the predicted SG is < 7.8 mmol/L (140 mg/dL) in 30 minutes and stop if < 4.4 mmol/L (79 mg/dL). AID challenges and opportunities related to exercise Regular physical activity and exercise are associated with numerous health benefits in PWD.

More specifically, for PWDs using AID systems during exercise, the possibility to optimize glycemia increases, particularly overnight. Considering the large percentage of factors that affect glucose control during and after exercise,^48^ the challenges are heightened, even with an AID system. Most studies on physical activity and type 1 diabetes, including AID systems, have been conducted in an adult population, but some have also been conducted in youth.^49,50^ Recently, a publication by Zaharieva et al^51^ summarized the practical aspects of using AID systems during exercise.

**Figure 1.**
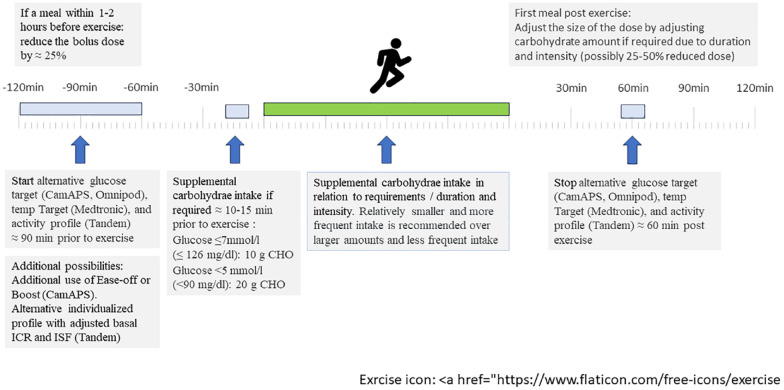
Summarized overview of current recommendations related to planned exercise.

Supplementary Material (Suppl. 1) highlights different exercise studies with various AID systems. Figure 1 summarizes the current recommendations related to planned exercise of varying intensities and durations and the practical strategies with these AID systems—all in the form of expert opinions.

## Discussion

This review highlights some of the positive outcomes related to AID technology use among youth with type 1 diabetes. In addition, this review describes some specific challenges that PWD and their parents/caregivers continue to face with AID technology, followed by possible solutions. Some situations must be approached differently, depending on the AID system used and the possibilities of manual individualized settings. In general, knowledge and ability are required to carry out the necessary adjustments with various AID systems. For example, insulin delivery may need to be temporarily increased or decreased in response to acute illnesses or exercise.—Moreover, the ISPAD consensus guidelines state that “It is recommended that youth be offered the most advanced insulin delivery technology that is available, affordable, and appropriate for them.”^12^ The term “affordable” in some countries may be related to the individual’s financial situation, while in other countries, it is about reimbursement via an insurance or national healthcare system, partially or fully paid by the state. When comparing an AID system with MDI treatment in combination with intermittently scanned CGM, health-economic advantages are described.^23,52^ An AID system could be seen as an investment rather than a short-term cost given the clear advantages.

The National Institute for Health and Care Excellence (NICE) recommends a target HbA1c level of 48 mmol/mol (6.5%) or lower for people with type diabetes or an individualized HbA1c level during pregnancy. At the same time, prevention of hypoglycemia is promoted. Consequently, the committee stated that AID systems should be recommended for children and young people with diabetes.^53^

It is essential to have well-designed clinical studies involving children in the future. Such studies might benefit from including the lateral effects seen via AID systems, ie, the impact on parents and siblings where sleep and general time gain are important outcomes besides analyses of health economic parameters, including cost-effectiveness.

In this review, including the supplementary material, we have added expert opinions regarding special situations that, from a clinical perspective, include distinct challenges. The various AID systems have some differences, generating varying solutions depending on the system used. To date, it has not been shown which of the AID systems works best in specific situations and the choice of treatment modalities are often influenced by the experience of the respective diabetes teams and users, rather than what has been evaluated in clinical studies with the different systems. The concept of “one size fits all” may, for some HCPs, offer simplicity, but the fact remains: person-centered care is what is being sought. A more diversified use of this diabetes technology consequently requires training of HCPs as well as PWDs. With this, we can achieve better clinical outcomes. Enhancing the user experience is essential, including tailoring diabetes devices for pediatric use and integrating psychosocial support into diabetes care models. Improvements to future AID systems, specifically for the pediatric population, may include improvements to specific components of the AID technology. New infusion sets must be developed to reduce the risk of skin problems related to adhesives. CGM systems need to be evaluated explicitly within a lower glucose and age range, including analyses of accuracy under different conditions (eg, during exercise or rapid changes in glucose). The AID system’s functionality and algorithms must be tailored and approved for use in individuals with lower insulin needs. Simplified processes are needed, including announced meals instead of carbohydrate counting or automated downloads to the desired platforms for analysis. A higher degree of actionable alerts may be a future consideration for this population. The regulatory perspective and the pathway for pediatric-specific devices required more attention to ensure equitable access for broader populations.

## Conclusion

Although glycemia may not be in the target range throughout every moment of the day, the AID systems represent a clear impact where not only does overall glucose improve significantly compared with MDI and CSII but so does overall quality of life. We continue to gain experience regarding the adaptations of the various AID systems and new approaches will be tested to meet several of the challenges that PWDs face. In addition, technological advancements continue to offer promise in the future.

## Supplemental Material

sj-docx-1-dst-10.1177_19322968241248404 – Supplemental material for Automated Insulin Delivery Systems in Pediatric Type 1 Diabetes: A Narrative Review Supplemental material, sj-docx-1-dst-10.1177_19322968241248404 for Automated Insulin Delivery Systems in Pediatric Type 1 Diabetes: A Narrative Review by Peter Adolfsson, Ragnar Hanas, Dessi P. Zaharieva, Klemen Dovc and Johan Jendle in Journal of Diabetes Science and Technology
