# Supplementary material for: Automated Insulin Delivery Systems in Pediatric Type 1 Diabetes: A Narrative Review
Source: J Diabetes Sci Technol. 2024 May 24;18(6):1324–33. doi: 10.1177/19322968241248404 (PMC11535396; doi:10.1177/19322968241248404)
Supplement: sj-docx-1-dst-10.1177_19322968241248404 – Supplemental material for Automated Insulin Delivery Systems in Pediatric Type 1 Diabetes: A Narrative Review [file sj-docx-1-dst-10.1177_19322968241248404.docx]

**Automated Insulin Delivery Systems in Pediatric Type 1 Diabetes: A Narrative Review**

**Supplementary material 2**

***Case 1***

**History:** A 16-year old female athlete with type 1 diabetes for four years and use of Dexcom G6 and Tandem t:slim X2 Control-IQ for 18 months. HbA1c 50 mmol/mol (6.7%) and no acute complications.

**Physical Activity:** Monday and Tuesday consist of two training sessions each. The first is a 30-min warm-up, 7.00-7.30 AM, followed by swimming 7.30-8.30 AM. In the afternoon, 30 min warm-up 4.30-5.00 PM, followed by swimming 5.00-6.30 PM.

**Diabetes Management:** She wakes up at 5.50 AM and takes insulin for her breakfast at 6.00 AM. She has the regular profile active before breakfast, reduces the dose by 75% and administers the same 10 minutes before the start of breakfast. She activates "Exercise Activity" directly, 45-60 min before warm-up starts, and otherwise adjusts using other predetermined, alternative profiles; plus 30/50 and minus 30/50, depending on glucose trends before exercise.

She adds a banana just before the warm-up starts and stops and disconnects the AID-pump before entering the swimming pool. She aims to avoid hypoglycemia and adds carbohydrates in relation to intensity every 20-30 min throughout the training. After the swimming session and a shower, she then starts and connects the pump again, usually with her everyday profile as this is adjusted to these conditions. During the afternoon training, a similar strategy is used.

**Figure 2.** Downloaded data to Glooko, including two exercise sessions highlighted in green.


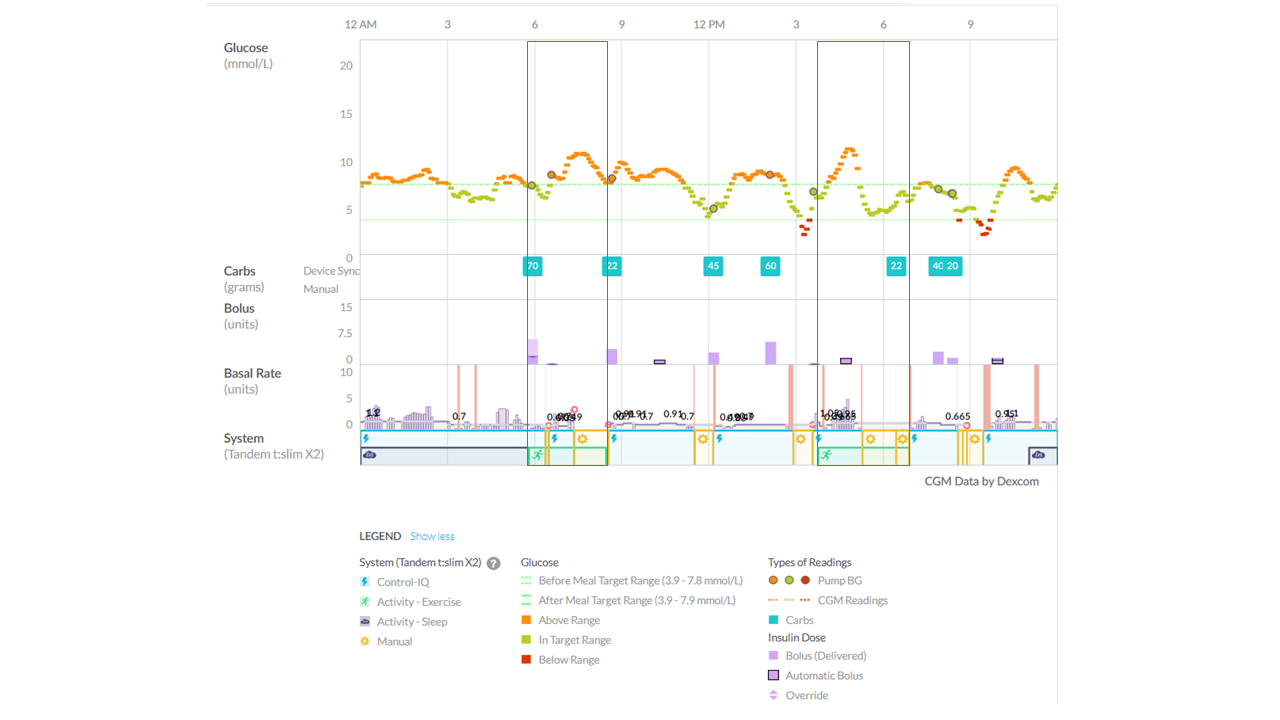


**Comment/Additional Considerations:**

The advice is to add “Exercise Activity” approximately 60 min before start of exercise and use additional profiles whenever needed. Normally we preset a minus 30 profile with ISF x 1.5 and a minus 50 profile with ISF x 2 compared to the everyday profile, if this is needed as possible, automated correction doses with this are gentler. However, due to the conditions, the Tandem t:slim X2 Control IQ pump needs to be stopped and disconnected during swimming.

***Case 2***

**History:** This is a 12 year old Non-Hispanic black male who has been living with type 1 diabetes for five years and has no acute or chronic complications. He is currently using the Omnipod 5 HCL system with a Dexcom G6 CGM for 10 months. His most recent HbA1c was 6.5% and his 4-week Glycemic Management Index (GMI) was 7.5% based on Dexcom Clarity reports.

**Physical Activity:** He plays soccer three times per week and games are usually once per week. The case below is from a game day. He started to warm-up with his team around 9:50-10:10AM and this included running laps around the field, dynamic warm-ups, sprints, passes, and shooting the ball. The game was from 10:15-11:30AM and he played the entire game. His position in soccer is left wing and striker.

**Diabetes Management:** For game day, he woke up around 8:30AM. He ate breakfast around 8:45AM and this consisted of two fried eggs with onions and 1 cup of whole milk. No insulin was administered (uncovered) for his breakfast in preparation for the soccer game.

He left the pump settings unadjusted for the game (i.e., he did not use Activity Feature). He drank water throughout the game and had no additional carbohydrates during the game.

Following the game, he went to eat pizza with his team. He ate approximately 100 grams of pizza and administered the full insulin dose around 20 minutes before the food arrived (i.e., pre-bolus). At 4:20PM, he had a 26 gram snack consisting of around eight crackers and the insulin bolus was administered around 2-minutes before consuming the snack. He ate dinner at around 6:00PM which consisted of pasta and meatballs with red sauce (74 grams, ~2 cups) and a second serving of pasta at 6:20PM (23 grams, ~2/3 cup). His insulin was administered at the start (i.e., no pre-bolus) of dinner.

**Figure 3.** Downloaded data to Glooko, including the soccer game highlighted in green.

**Comment/Additional Considerations:**

This child has done a great job managing his glycemia during his soccer game. Since his breakfast contained limited carbohydrates (~15g), choosing to have breakfast as an uncovered meal (i.e., no bolus insulin) was helpful in keeping glycemia more stable and limit the drop pre-exercise. Consensus guidelines commonly recommend setting Activity Feature well in advance of exercise (1-2 hours pre-exercise) until the end of activity (REF). However, in many cases such as this one, he forgot to use Activity Feature, but glycemia remained stable throughout the game. We would also continue to encourage pre-bolusing for meals and snacks. When eating a high carbohydrate and high fat meal such as pizza, we often recommend and extended bolus, but this option is not available with Omnipod 5 in automated mode. Another option would be to switch the pump to Manual mode and enter the carbohydrates, CGM value, and choose “Extend Bolus”. The amount of insulin delivered can be adjusted so the bolus insulin delivery is spread out over a duration of time. Post-exercise, considerations include pre-bolusing for snacks and dinner since most of the hyperglycemia that occurred was after the snack and dinner. If hypoglycemia commonly occurs with other activities (e.g., soccer practice versus a soccer game), another system that may work well for this child would be the CamAPS system where the “planned ease-off” feature can be programmed in advance to automatically start 1-2 hours before activity.

**Case 3:**

**History:** This is a 10 year old Non-Hispanic white girl who has been living with type 1 diabetes for two years with no diabetes-related complications. She has been using the Omnipod 5 HCL system and Dexcom G6 CGM for five months. Her most recent 90-day GMI was 7.3% with a mean glucose of 165 ± 56 mg/dL.

**Physical Activity:** She plays basketball twice per week as well as dance and horseback riding once per week. The case below is from a day where she had basketball practice from 6:30-7:30PM. She started basketball warm-ups around 6:30PM and this included running laps around the court, dynamic warm-ups, dribbling the ball, and layups. Practice was fast-paced with a rating of perceived exertion (RPE) of 8. Her practice involved drills including layups, followed by a game of scrimmage (i.e., running and playing basketball).

**Diabetes Management:** She woke up around 6:30AM and took her insulin for breakfast at around 7:50AM (pre-bolusing around 15 minutes before eating). Her breakfast consisted of ~45g of cereal and milk, as well as avocado toast and fruit. She arrived at school around 8:20AM and had recess at 10:00AM. She had an apple juice (~14g) at recess and was not active. She had lunch around 12:00PM (pre-bolusing 10 minutes before eating) and she ate half of a bagel, a granola bar, and four slices of apple (~51g). She had a few snacks afterschool including at 3:10PM (chips and an apple; ~35g), at 4:07PM (peanut butter and Nutella sandwich with two pieces of bread; ~26g), and at 5PM (a granola bar; ~10g). She set Activity Feature at 5:00PM for three hours (until 8:00PM) and had a gummy candy with no bolus on the way to practice ~6:15PM.

Her dinner after basketball was at 8:50PM and she ate ~35g of carbohydrates (no pre-bolus). She ate 1 medium-sized baked potato with cheese, bacon and sour cream, pesto chicken, and oven-roasted broccoli. She also had a snack after dinner that included yogurt and fruit (~28g).

**Figure 4.** Downloaded data to Glooko, including the soccer game highlighted in green.

**Comment/Additional Considerations:**

In preparation for basketball, this case shows that she did a wonderful job managing her blood glucose levels throughout practice. As suggested by the ISPAD exercise clinical consensus guidelines (REF), it is recommended to set a higher exercise target (Activity Feature) at least 1-2 hours pre-exercise until the end of exercise, just as she had done. In her case, she also kept Activity Feature on for 30-minutes post-exercise. As seen in the evening post-exercise and following dinner, she experienced hypoglycemia. A recommendation would be to reduce the insulin bolus by ~25% due to the increased insulin sensitivity post-exercise. An additional recommendation throughout the day would be to try and pre-bolus 15-20 minutes in advance of larger meals to reduce the likelihood of a glucose spike.

***Case 4***

**History:** A 19-year-old male has been living with type 1 diabetes for seven years and there were no acute medical complications. For the past two years he has been using the MiniMed 780G system and his most recent 30-day TIR was 78%, TBR was 3% and GMI was 6.6%. Two years after diagnosis he started to be an active cyclist (both mountain biking and road cycling) and for the past two years he was engaged in a regular training process. In the last year, he conducted on average approximately four activities per week (214 activities in total) with a mean distance of an activity of 54.5 km and average activity duration of 2.4 h.

**Physical Activity:** The case below is from a longer mountain-bike ride day. He started his ride at 10:20 AM. Total moving duration time was 4 h and 15 min with a total distance 102 km and elevation gain 1428 meters. Average weighted power was 211 W (maximum 573 W) and total spent energy was estimated at 2988 kJ.

**Diabetes Management:** For the ride day, he had breakfast at around 8:30 AM with approximately 0.75 g of carbohydrates per kilogram of body mass and a full (unadjusted) insulin bolus was given. He ate a small snack (21 g of carbohydrates) one hour before the ride and again a full insulin bolus given. He consumed additional 45 g of simple carbohydrates (gels, sugared beverages) during the training that were uncovered. At the end of the ride, he ate 25 g of carbohydrates, again without any insulin dose adjustment (full bolus was given).

He set his temporary target (activity mode) after he started to ride, and the total duration of this setting was around three hours.

**Figure 5.** Downloaded data to CareLink (and Strava), including the mountain-bike ride highlighted in red.


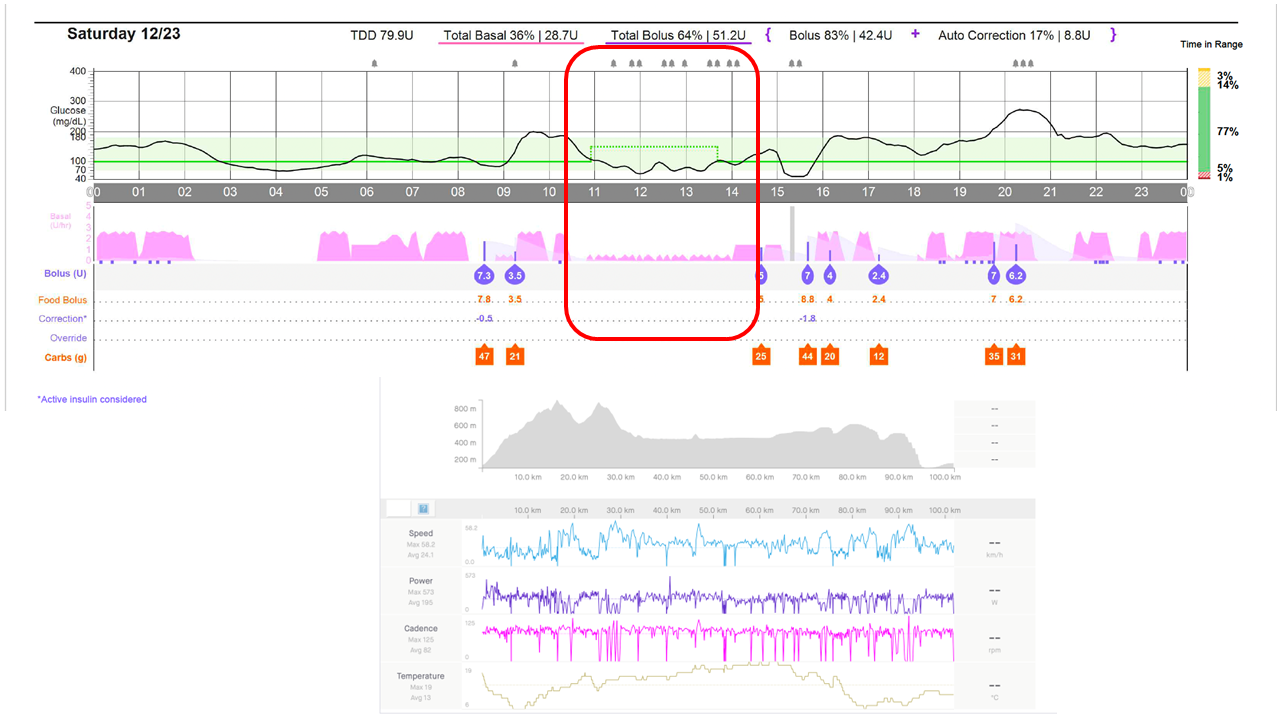


**Comment/Additional Considerations:**

As seen in the glucose profile (Figure 3), his sensor glucose levels were in the lower range for the entire ride reaching hypoglycemia range on several occasions. He correctly did not cover 45 g of carbohydrates during the training. Additionally, he had a hypoglycemic event following meal after the ride. The advice would be to adjust carbohydrate coverage for the meal before and after the training (25-50% reduction of insulin bolus).

Furthermore, we would recommend to use temporary target feature (activity mode) well in advance of the ride (1.5-2 h pre-exercise) until the end of activity (or even 15-30 min post activity). Importantly, we would suggest nutritional consultation to recommend the amount of carbohydrates consumed during the prolonged activity (ranging from 0.4 to 1.3 g carbohydrate per kg of body mass per hour) to enhance performance and to prevent hypoglycemia ^34^.

The four above cases show some of the similarities seen between the different AID-systems but also some differences.
